# NEIGHBORHOOD CONDITIONS AND PERSONALITY CHANGE AMONG OLDER ADULTS

**DOI:** 10.1093/geroni/igac059.2377

**Published:** 2022-12-20

**Authors:** James Iveniuk, Kathleen Cagney

**Affiliations:** NORC at the University of Chicago, Chicago, Illinois, United States; University of Michigan, Ann Arbor, Michigan, United States

## Abstract

Conscientiousness is a key aspect of personality, associated with overall better health and wellbeing across the life course, including among older adults. The past ten years have seen a growing interest in how social-environmental factors impact personality traits, but the impact of neighborhood conditions is rarely investigated. Broken windows theory argues that changes to residents’ character can be brought about through signals of disinvestment in local norms (i.e. visible disorder), pointing to neighborhood disorder as a possible environmental condition that can shape personality. Drawing upon three rounds of data from the National Social Life Health and Aging Project (2005/2006, 2010/2011, 2015/2016; N=1554), we investigate longitudinal associations between traits and changing neighborhood conditions using a Big Five self-assessment, and interviewer ratings of local disorder. Using a combination of mediation and cross-lagged models, we find mixed support for broken windows perspectives – significant mediation processes, but no cross-lagged effects.

